# Synthesis, Characterization, and Applications of Silk/Bentonite Clay Composite for Heavy Metal Removal From Aqueous Solution

**DOI:** 10.3389/fchem.2019.00654

**Published:** 2019-10-09

**Authors:** Nasira Wahab, Muhammad Saeed, Muhammad Ibrahim, Akhtar Munir, Muhammad Saleem, Manzar Zahra, Amir Waseem

**Affiliations:** ^1^Department of Chemistry, University of Kotli Azad Jammu and Kashmir, Kotli, Pakistan; ^2^Analytical Lab, Department of Chemistry, Quaid-i-Azam University, Islamabad, Pakistan; ^3^Department of Chemistry and Chemical Engineering (SBASSE), Lahore University of Management Sciences, Lahore, Pakistan; ^4^Department of Chemistry, Lahore Garrison University, Lahore, Pakistan

**Keywords:** silk fibroin, bentonite, adsorption, heavy metals, environmentally friendly

## Abstract

Bentonite clay is an abundant and low-cost adsorbent and silk fibroin, a naturally occurring protein, and both have a low capacity to remove lethal heavy metal ions from aqueous solution separately. To enhance their metal adsorbing capacity, a new silk fibroin-based bentonite composite was prepared for improving water quality by eliminating heavy metal ions i.e., lead, cadmium, mercury, and chromium. The as-synthesized composite shows better metal sorption capacity than either of them alone. To analyze their structural properties and characteristic functional groups, X-ray diffraction and Fourier-transform infrared spectroscopy were used. The specific surface area for silk/bentonite composite was about 4 m^2^/g that is smaller than the unmodified bentonite (23 m2/g) which indicates the impregnation of bentonite onto the silk fibroins. Scanning electron microscopy results shows the changes in morphology from plate aggregates to rosette like arrangements. The XRD results of clay/composite shows an increase in basal spacing (d_001_, from 1.55 to 3.34 nm) in comparison to pristine clay. FTIR results show the presence of organic moiety in SF clay composite. The mechanism of adsorption based on complex formation and ion exchange were proposed briefly. Various adsorption isotherms and kinetic models were applied for the removal of Pb(II), Cd(II), Hg(II), and Cr(VI). As the kinetic study was concerned, kinetic data fitted well to pseudo second order kinetics because experimental values of q_e_ are much closer to the calculated values. The adsorption equilibrium was best studied by Langmuir isotherm whose regression coefficient values (0.985–0.995) are best when compared to Freundlich adsorption isotherm (0.954–0.990) and are indicative of homogeneity of adsorption sites on the SF/clay composite. The monolayer adsorption capacity for Cd(II), Pb(II), Hg(II), and Cr(VI) was found to be 11.35, 11.1, 10.5, and 10.2 mg/g, respectively.

## Introduction

Silk (*Bombyx mori)* is a naturally occurring polymer made up of fibroin and glue like protein sericin, that holds the fibroins together (Zhang et al., 2009). Silk fibroin consists of two polypeptide chains, a heavy H-chain (390 kDa) and a light L-chain (26 kDa) these both are connected by disulphide bond forming H-L complex (Qi et al., 2017). A glycoprotein (P25) is likewise non-covalently associated with the H– L complex. All three together (L-chain, H-chain, and P25) appeared as 6:6:1 proportion in silkworm to frame silk. SF made from amino acids essentially of Ala (30%), Gly (43%), and Ser (12%) in appropriate proportion. There is a complex hexapeptide sequence of amino acids Gly-Ala-Gly-Ala-Gly-Ser in its hydrophobic regions, whereas, H-chain shows a repeat of Gly-Ala/Ser/Tyr dipeptides. L-chain carries non-repetitive arrangements of amino acids and is more hydrophilic (Mieszawska et al., 2011). Silk fibroin has gained much importance in the formation and development of novel material because of its higher reactivity, mechanical properties, biodegradability, and biocompatibility (Kishimoto et al., 2013). The statistical data shows that in twenty-first century global silk cocoon production is >170,000 metric tons (Kwak et al., 2016). In recent years, natural protein fibers has been explored for novel type of sorbent production because of their metal absorption capacity (Goto and Suyama, 2000).

Silk (*Bombyx mori)* possess many ionizable groups on different amino acid residues on the side chain and its separation depends on the value of pH in surrounding medium as it is amphoteric in nature. Free carboxyl groups of different acids that occur in the polypeptide sequences are glutamic and aspartic acid are the sites for binding of SF with metal ions (Taddei et al., 2003). The pKa value of these groups ranges from 4 to 4.8 and detached completely at pH = 7 and negatively charged groups are present to bind metal ions (Maclaren and Milligan, 1981). Fibrous composition of natural silk has limited applications; therefore, it is fundamental to break up silk fiber and recover it. However, the recovered silk protein materials demonstrate minor mechanical properties and greater unsteadiness. To minimize the instability of the SF films and to obtain the material with good strength and more flexibility SF materials are combined with some suitable reinforcing fillers (Dang et al., 2010).

Clays are for the most part hydrous aluminosilicates which comprise of blends of fine grained earth minerals, metal oxides, and precious stones of different minerals (Demiral et al., 2008). Bentonite consists of montmorillonite with composition of Al_2_O_3_, MgO, CaO, SiO_2_, K_2_O, and Fe_2_O_3_ (Ngah et al., 2011). The 2:1 layer structure of bentonite comprise of an octahedral alumina sheet fit in the two contradicting tetrahedral silica sheets. The thickness of these layers are in few nanometers range and length is several microns and arranged into stacks with the gap filled with exchangeable metal cations (Alexandre and Dubois, 2000).

Bentonite is used as an adsorbent for removal of metal ions because of its cation exchange capacity, larger surface area and adsorptive capacity for different organic and inorganic ions (Donat et al., 2005). Besides various applications of clay composites (Munir et al., 2019), bentonite clay is more hydrophilic, in order to increase its adsorption capacity and surface modification, usually organic molecules (most often surfactants) is used to transform its properties from hydrophilic to more hydrophobic (Nafees et al., 2013; Nafees and Waseem, 2014; Ullah et al., 2017). Because of the hydrophobic nature of organoclays, this filter material can be used for purification of water (Xi et al., 2010). Although clay possesses the ability of adsorption of various hydrophilic substances, the modification of their surface can successfully enhance their capabilities (Liu, 2007; Monvisade and Siriphannon, 2009). In these inorganic-organic hybrids, mostly inorganic minerals are deposited in organic matrix by covalent crosslinking or hydrogen bonding. Formation of these hybrids is advantageous because of low density, high flexibility, and toughness of polymers and excellent mechanical properties (Hou and Chen, 2010). That is why the modification of bentonite by using silk fibroin polymer was performed to achieve better sorption capacity for heavy metals. The detailed discussion on the conformational features and morphology of SF/clay nanocomposites have been reported previously (Dang et al., 2010; Kishimoto et al., 2013).

A large variety of heavy metals are involved in industrial processes and the discharge of these heavy metals causes a severe environmental problem as these metals are water soluble and accumulated quickly in living bodies. Higher concentration of different heavy metals, Cd, Cr, Cu, Hg, Pb, Zn adversely affect the living body as these are typically hazardous metals (Waseem et al., 2014; Ahmed and Ahmaruzzaman, 2016; Waseem and Arshad, 2016). Various adsorbents have been reported in literature for the removal of heavy metals such as Strychnos potatorum seeds (Senthil Kumar et al., 2013), sulfuric acid-modified eucalyptus seeds (Pearlin Kiruba et al., 2014), nano-scale zero-valent iron impregnated cashew nut shell (Kumar et al., 2011; Prabu et al., 2015), coffee waste based carbons (Suganya and Senthil Kumar, 2018), dried algal biomass (Gunasundari and Senthil Kumar, 2017, chitosan blends (Anitha et al., 2015; Liang et al., 2019), TiO2/montmorillonite clay composites (Dou et al., 2011). The composites of silk with montmorillonite were also reported that are prepared to use in bone regeneration (Kishimoto et al., 2013). In this study we have prepared an SF/bentonite composite that is a new candidate for the adsorption of heavy metal ions from aqueous solution. SF and bentonite make strong hydrogen bonding due to large number of available H-bonding sites on SF and terminated O-sites on the clay. To the best of our knowledge, this is the first attempt to use SF/bentonite clay composite for the sorption of heavy metal removal.

## Experimental

### Materials and Methods

All other chemicals used were of high purity and used without further refining. Silk cocoons were very kindly provided by the Agricultural Department, Kotli, AJ&K. Bentonite obtained from Alfa Aesar (Karlsruhe, Germany).

### Preparation of Regenerated Silk Fibroins

*Bombyx mori* silk cocoons were cut into smaller pieces and then degummed by using 0.05 M aqueous solution of Na_2_CO_3_ for 1 h and then rinsed thoroughly by using distilled water to remove glue like protein sericin. Then the degummed fibers were dried at 50°C. These degummed fibers were named as SF. These degummed fibers were then dissolved in aqueous solution of 9 M LiBr at room temperature to obtained SF aqueous solution that was then dialyzed with distilled water for 96 h by using slide-a-layer dialysis cassettes to remove salt. This dialyzed solution was centrifuged at 5,000 rpm for 30 min (Dang et al., 2010). The supernatant was collected and dried at 50–70°C. The dry powder that obtained was named as SF1.

### Preparation of SF/Bentonite Composites

SF/Clay nanocomposites were prepared by modifying the method reported previously (Dang et al., 2010) by adding 2 gram of bentonite clay in 50 ml distilled water and ultrasonicated it for 30 min to improve dispersion. Then its pH is maintained to 3 by adding few drops of HCl. The dry silk fibroin powder was then added to about 2 gram and maintain its stirring over night at 50°C. After stirring dried the sample in an oven. The dried sample was then removed and grinded into fine powder that are named as B-SF.

### Instrumentation

The prepared composite was analyzed using Fourier Transform Infrared (FTIR) spectrophotometer (BRUKER-TENSOR-27) in the range of 4,000–400 cm^−1^ having scan rate of 15, with the resolution of 4 cm^−1^. Powder X-ray diffraction pattern of unmodified bentonite (RBT), silk fibroin (SF), and modified bentonite (BSF) were performed by using XRD Model No. D8 Advance Bruker with Cu-Kα (λ) 0.154 nm with scanning rate 2° in the range of 2θ = 5–70°, step size 0.025, step time of 0.4 s, and scan speed of 0.06/sec. The morphology of scaffolds was examined by scanning electron microscopy (JEOL JSM-5910) at 10 kV and 10 Pa under low vacuum mode. The pore size and BET surface area of samples were analyzed by (Micrometrics, ASAP 2020) System and samples were degassed for 12 h at 70°C.

### Adsorption Procedure

A modified clay batch method was used to study the adsorption of heavy metals on silk. In this experiment, stock solutions of different metal salts such as lead nitrate, mercuric nitrate, cadmium acetate, and potassium chromate were prepared in 0.1M nitric acid. These stock solutions are used to prepare other solutions of desired concentrations for adsorption experiment in deionized water. A known quantity of adsorbent with 25 ml of aqueous solution of different heavy metal salts whose concentrations are known is kept on a hot plate for stirring for a fixed time. After stirring, the solutions were removed and centrifuged to remove the composites. The residual metal ions concentration in reaction mixture were determined by using Agilent Technologies 55 AA atomic absorption spectrometer. The effect of different experimental factors like pH, initial concentration of metal ions, amount of adsorbent, contact time, and temperature on adsorption of heavy metals by silk/clay composite were studied by using Atomic absorption spectrometer.

To study the heavy metal ions adsorption on SF/bentonite composites, different parameters were applied such as pH, effect of contact time, amount of adsorbent, concentration of heavy metal solution, and effect of temperature on heavy metal ions adsorption.

The percentage efficiency (S%) and adsorption capacity at equilibrium for adsorption of analyte were determined by following Equations (1) and (2);

(1) $$Sorption\;Efficiency\;\left(S\%\right)=\frac{\left(Ci-Ce\right)}{Ci}\times 100$$

(2) $$qe=\frac{\left(Ci-Ce\right)}{m}xV$$

Where qe is the adsorption capacity (mg/g), Ci and Ce are the initial and equilibrium concentrations of the analyte (mg/ dm^3^), respectively, V is the volume (dm^3^) and m is the mass of adsorbent (g).

## Results and Discussion

### Characterization

#### FTIR Analysis

**Figure 1:**
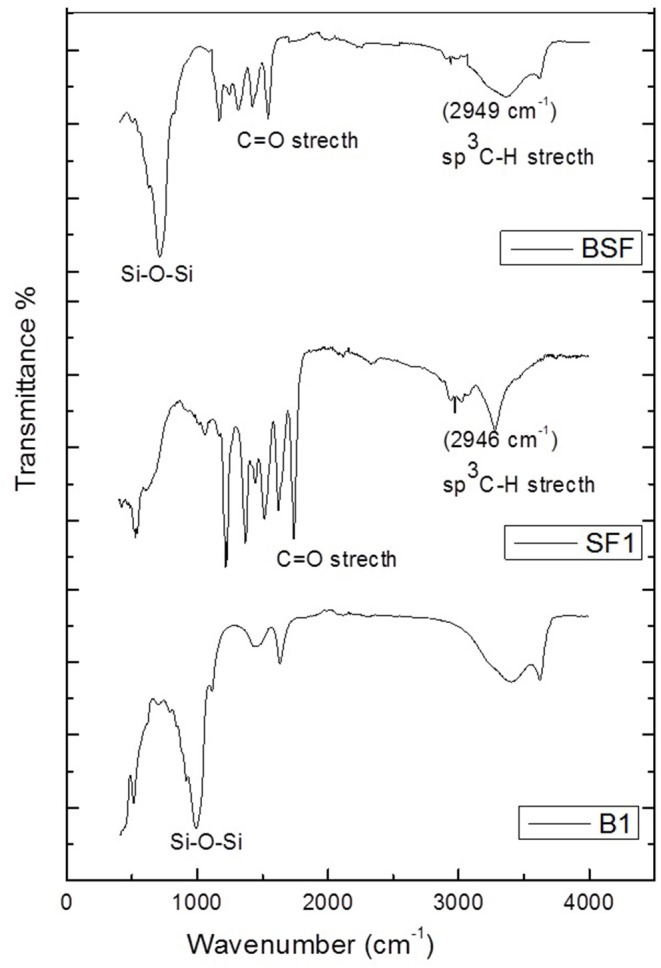
FTIR Spectra of silk fibroin (SF1), bentonite clay (B1) SF/Clay composite (BSF).

In the FT-IR spectra of silk fibroin (Figure 1), three different types of peaks are associated with amide groups in various proteins. The characteristic peak observed at 1738 cm^−1^ because of the stretching vibration of (C=O). The peaks observed at 3,275 and 1,621 cm^−1^ corresponds to stretching vibration and bending vibration of N-H, respectively. The peaks appear in spectra at 1,216 and 1,228 cm^−1^ shows the stretching vibration of C-O and C-N, respectively. The presence of amide-I, amide-II and amide-III can be observed by the vibrational bands at 1,620 cm-1, 1,517 cm^−1^, and 1,229 cm^−1^, respectively (Kishimoto et al., 2013). In the spectra of silk/bentonite composite (B-SF), the characteristic peaks appear at 1,365 and 1,428 cm^−1^ and at 2,960 and 3,010 cm^−1^ indicate the presence of (sp^3^ C-H) and (sp^2^ C-H) due to CH_2_ and CH_3_ bending and stretching, respectively, which are absent in simple bentonite spectra (RBT). The peaks appear at 1,738, 1,644 cm^−1^ corresponds to C=O stretch and N-H bending, respectively, which are also absent in bentonite spectra. The band observed in the spectra of bentonite at 3,404 cm^−1^ is due to OH stretching of hydroxyl groups and water present in the mineral and at 1,632 cm^−1^ is due to OH bending vibration. The band observed at 913 cm^−1^ is due to OH deformation mode of Al-Al-OH or Al-O-Al and at 990 cm^−1^ is Si-O-Si stretch which are also observed in the spectra of silk/bentonite composite with certain modification (Hou and Chen, 2010). These peaks confirm the formation of silk fibroin/clay composite.

#### XRD Analysis

**Figure 2:**
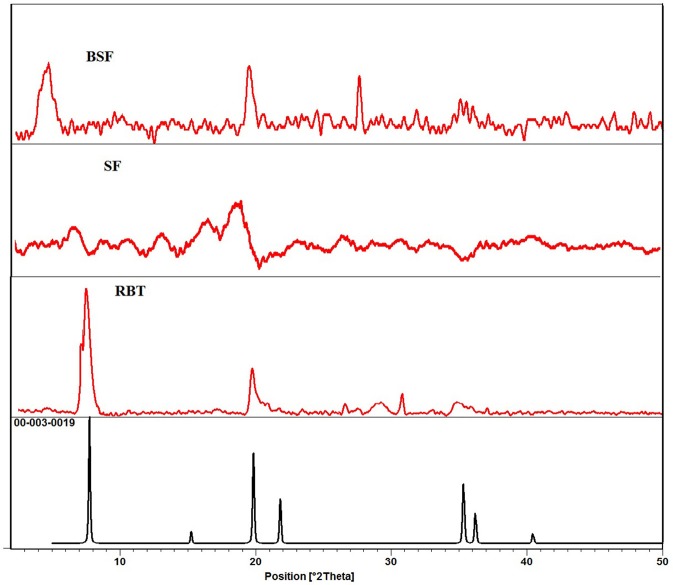
XRD spectra of bentonite (RBT), silk fibroin (SF), and SF/bentonite composite (BSF).

In XRD spectra of bentonite (RBT), the diffraction peak observed at (2θ = 5.67° and 19.69° which have basal spacing of 15.56 and 4.50 Å with relative high intensity of (001) and (020) plane, respectively, matches well with the JCPDS reference code 00-003-0019. The reflection at (2θ = 61.82°) which has basal spacing 1.50Å shows the di-octahedral structure of bentonite. The peaks at 2θ = 19.69°, 26.63°, and 29.16° with basal spacing 4.50, 3.34, and 3.06 Å indicate the presence of quartz (SiO_2_) (Figure 2). The other peaks are due to impurities in natural bentonite. In this study, all the peaks appears in the unmodified bentonite are same as in silk/bentonite composite spectra except the peak at 2θ = 6.28° with d-spacing (1.55 nm) in unmodified spectra (RBT) is disappear in the silk fibroin based modified bentonite (BSF) and appeared at 2θ = 3.04° with d-spacing (3.34 nm), which concludes that the d-spacing of silk fibroin based modified bentonite is expanded during composite formation of SF/clay. The increase in d-spacing is a usual indicator of intercalation of organic moiety in to the structure of clay (He et al., 2006; Wang and Wang, 2008).

#### SEM and BET Analysis

**Figure 3:**
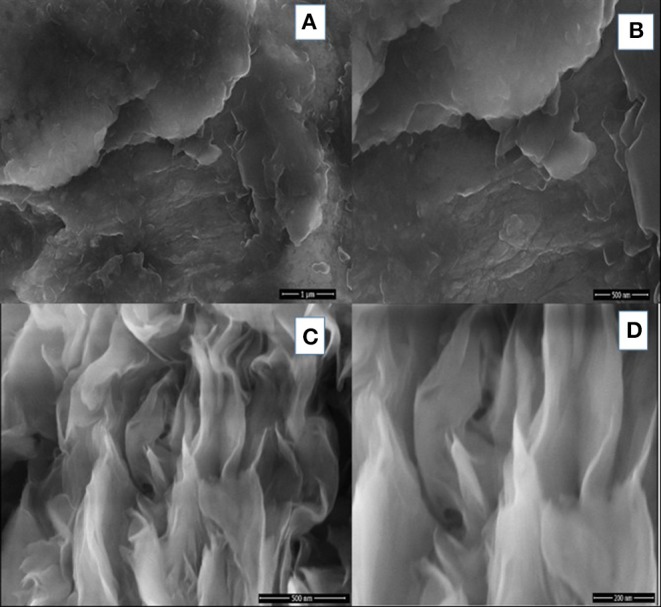
SEM images of Unmodified clay **(A,B)** and modified SF/clay composite **(C,D)** at different resolutions.

**Figure 4:**
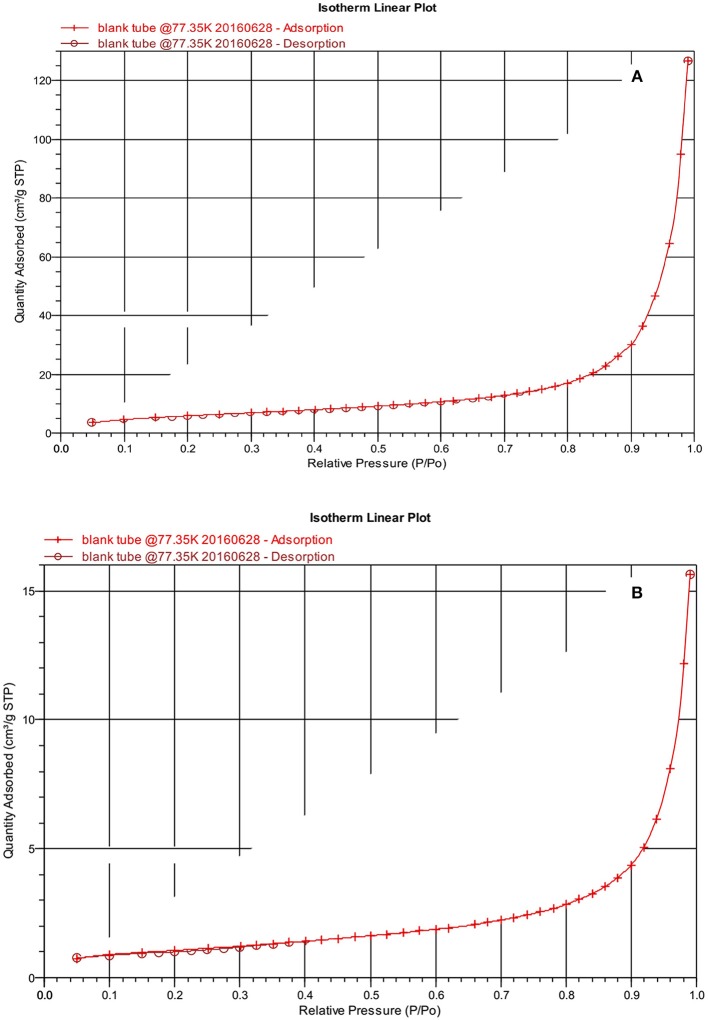
BET isotherm of surface area of pristine clay **(A)** and SF/bentonite composite **(B)**.

To analyze the surface morphology of the prepared nanocomposites, SEM technique is used. The surface morphology of bentonite clay and SF/clay composite are shown in Figure 3. For this reason, cross sectional views were taken which showed the layered structure with spacing among layers. Difference in morphology of unmodified bentonite and silk/bentonite composite are clearly shown by SEM images. The flakes and rosette like arrangement of silk fibers along with bentonite particles were shown in Figure 3C. This arrangement resulted in the increased adsorption capacity owing to the appearance of more active sites for adsorption. The BET surface area for silk/bentonite composite was about 4 m^2^/g and that is smaller than the unmodified bentonite (23 m^2^/g) which indicates the impregnation of bentonite onto the silk fibroins. The specific surface area decreased probably due to clay particle surface covered by SF (Figure 4). The decrease in surface area also results from the packing density of the organic moiety into the interlayer of clay. This sort of reduction in surface area is usually occurred in organically modified clays as reported previously (Wang and Wang, 2008; Ullah et al., 2017; Saeed et al., 2019).

#### Effect of pH on Adsorption Efficiency

**Figure 5:**
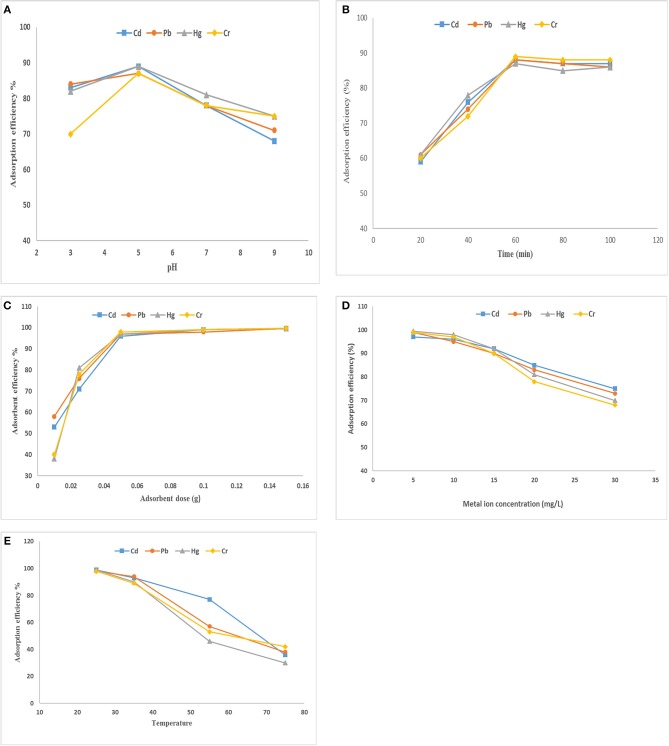
Effect of **(A)** pH, **(B)** contact time, **(C)** adsorbent dose, **(D)** metal ion concentration, **(E)** temperature on adsorption of heavy metals by SF/bentonite composite.

During the process of adsorption, the pH of aqueous solution was considered very important because it affects the surface properties of the adsorbent. The effect of pH on the adsorption capacity of SF/bentonite composite for heavy metal ions such as Cd(II), Cr(VI), Cu(II), Hg(II), Pb(II), Zn(II) adsorption tests were done by taking initial concentration of 10 mg/L of chromium, cadmium, lead, and mercury and 0.05 amount of adsorbent was taken using 30 min as contact time and by changing the pH of solution in range of 3–9. The effect of pH change on adsorption of Cd(II), Cr(VI), Cu(II), Hg(II), Pb(II), Zn(II) is shown in Figure 5A. The results show excellent removal capacity of these heavy metal ions by SF/clay composite. It is clear from the figure that by increasing pH of solution adsorption of heavy metal ions decreases. At higher pH the precipitation of metal ions started and metal hydroxides produced (Anirudhan et al., 2012) which prevents normal working on them. At pH = 5 the ion exchange and the complex formation process are the main procedure to remove metal ions from the solution. So, the pH = 5 was adjusted to perform further optimization experiment.

#### Effect of Contact Time on Adsorption Efficiency

Contact time effect on adsorption of heavy metals by SF/clay composite was also studied by taking initial concentration of 10 mg/L for Cd (II, Pb (II), Hg (II), and Cr (VI) ions, respectively. Contact time required by maximum adsorption of these ions is represented in Figure 5B. There is an increase in adsorption at initial stage and maximum adsorption observed at first 60 min, beyond that, increase in adsorption was slowed down and gained equilibrium as there is no change in adsorption was observed. The increase in adsorption observed at initial stage might be due to large number of available binding sites, higher surface area, availability of pores and adsorption sites are well-exposed (Gandhi et al., 2014). The sorption efficiency observed for Cd (II), Pb (II), Hg (II), and Cr (VI) ions was 83, 79, 91, and 88 percent, respectively. As a result, stirring time was fixed at 60 min to attain the adsorption equilibrium in rest of the experiments.

#### Effect of Adsorbent Dose on Adsorption Efficiency

To study the effect of adsorbent dose on the heavy metal ions removal, solution of heavy metal salt of various concentration was taken while the amount of SF/bentonite adsorbent was taken in range 0.01–0.15 mg and shaking for 60 min. The results that are represented in Figure 5C. It is indicated that by increasing the amount of adsorbent, adsorption efficiency also increased due to increase in the number of adsorption sites available and greater surface area to adsorb metal ions. Greater availability of exchangeable sites enhanced the metal uptake (Tomul and Basoglu, 2010).

#### Effect of Metal Ions Concentration on Adsorption Efficiency

In order to explain the effect of initial concentration of metal ions on the adsorption process, the solution of metal salts of different concentrations, such as 1–30 ppm of Cd(ll), Pb(ll), Hg(ll), and Cr(VI) was prepared and stirred for 60 min by adding 0.05 g adsorbent. Mostly, the adsorption percentage decreased as the concentration increased (Figure 5D). This is because at low concentration, the ratio between numbers of metal ions to available adsorption sites is small as a result adsorption occurs without depending on concentration but when concentration increases competition between metal ions for adsorption site increased. Hence, adsorption decreases but value of q_e_ increases (El-Maghrabi and Mikhail, 2014).

#### Effect of Temperature on Adsorption Efficiency

To study the effect of temperature on heavy metal ion adsorption, a solution of heavy metal salt of cadmium, lead, chromium and mercury was taken along with 0.1 gram of adsorbent with constant stirring for 60 min at temperatures of 20°, 40°, 60° and 80°C. The results obtained are described in Figure 5E, which indicates that the adsorption efficiency is maximum at lower temperature and it is about 92, 93, 85, and 88 percent for Cd(ll), Pb(ll), Hg(ll), and Cr(VI), respectively. The decrease in adsorption percentage by increasing temperature is because of increase in available thermal energy that causes desorption. The mobility of adsorbate is higher at higher temperature that causes desorption (Gandhi et al., 2014).

### Adsorption Mechanism

**Figure 6:**
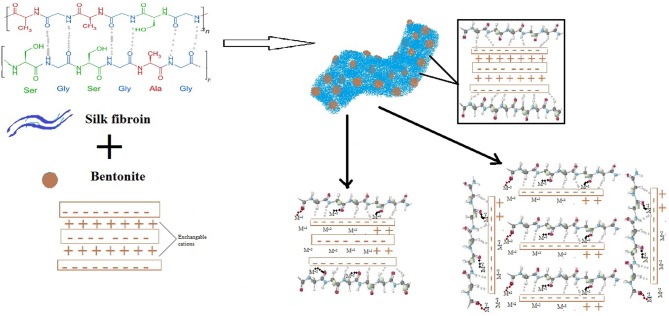
The arrangements of SF and clay in SF/clay composite.

SF can be found in three different structural forms, i.e, random coil, alfa-helix and beta sheets, usually beta-sheet structure is required to achieve better strength, so the formation of these sheets is important during composite formation (Dang et al., 2010). In the present study, the formation of SF/Clay composite yielded mixture of random coils and beta sheets as reported previously (Dang et al., 2010). The presence of β-sheets of SF on F/Clay composite provide more available amino-acid groups to be required to bind with metals than alone in SF or clay. The random coils of SF alone mainly attached with strong hydrogen bonds, which after modification and dispersion with clay (β-sheets content increases) results better arrangement for metal ion binding. The possible arrangements that SF can orientate with clay is given in Figure 6, where β-sheets were shown to interact with clay making random stacked arrangements, clay sheets provide the support to random coil to straighten and provide more room to chelate with heavy metals.

The formation of metal ion complex was brought about by the interaction between metal ions and carboxylic functional groups of amino acid residues present in silk fibroin (Ki et al., 2007). Through the coordination of hydrated metal cations with amino and carboxylic groups, a metal chelates resulted. The amino group has a lone pair that it can share with metal cations. In this study, the SF/clay composite provided extra binding sites for the binding of metal cations, and ion exchange sites and increased surface area was available for adsorption. When in metal ion solutions, this composite was dispersed, and interaction between silk fibroin and clay occurred for adsorption of metal ions through an electrostatic interaction which developed between an acidic group of amino acids and the metal ions. In the meantime, interlayer cations of bentonite might be replaced by heavy metal ions. This effect modifies the removal of heavy metal ions by SF/bentonite composite. To check the synergistic effect of SF with clay, individual studies were also performed under the optimized conditions. Table 1 depicts that the best results shown by the SF/clay composite than either of SF or bentonite clay. Table 2 compares the monolayer adsorption capacity of various modified and unmodified absorbents used individually for various heavy metal ions reported in literature with the present study.

**Table 1:** The comparison the metal adsorption capacity of SF, clay, and SF/clay composite.

**Metal ions**	**Commercial bentonite q_**m**_ (mg/g)**	**SF q_**m**_ (mg/g)**	**SF/clay composite q_**m**_ (mg/g)**
Cd(II)	1.1	1.5	11.35
Pb(II)	1.9	1.3	11.1
Hg(II)	1.8	1.4	10.5
Cr(VI)	1.2	1.7	10.2

**Table 2:** The comparison of adsorption capacity (q_m_) of various reported adsorbents.

**Adsorbent used**	**Modification**	**Cd(II) (mg/g)**	**Cr(VI) (mg/g)**	**Hg(II) (mg/g)**	**Pb(II) (mg/g)**	**Reference**
Cashew nut shell	–	22.11	–	–	–	Kumar et al., 2012
Surface modified Eucalyptus seeds	Sulfuric acid HCl	71.1564.16	–	–	–	Kiruba et al., 2014
Rice husk	–	21.28	–	–	–	Kumar et al., 2010
Pure smectite (clay)	–	3.87	–	–	–	Bedoui et al., 2008
Coffee waste based carbons	Ultrasonic assisted active carbon	–	156.7	–	–	Suganya and Senthil Kumar, 2018
Ectodermis of Opuntia-protonated	Sulfuric acid	–	3.47	–	–	Barrera et al., 2006
Chitosan	2,6-diaminopyridine	–	–	172.7	–	Liang et al., 2019
TiO2/montmorillonite	–	–	–	123.8	–	Dou et al., 2011
Strychnos potatorum seeds	Sulfuric acid	–	–	–	166.67	Senthil Kumar et al., 2013
Cashew nut shell	Sulfuric acid	–	–	–	71.15	Kumar et al., 2011
Chitosan–blends	Polyacrylonitrile	–	–	–	20.08	Anitha et al., 2015
Clay-silk blend	–	11.35	10.2	10.5	11.1	Present study

### Adsorption Isotherm Models

To determine the equilibrium adsorption data, various isotherm models can be used. In the present work, a Langmuir and Freundlich model were used to represent the relationship among the amount of adsorbed metal ions and their initial concentration at a constant pH.

### Langmuir Adsorption Isotherm

According to Langmuir adsorption, the isotherm adsorption occurs at a definite homogeneous position in the adsorbent and indicates the monolayer adsorption. The linear form of Langmuir isotherm is expressed as:

$$\begin{array}{l} \frac{C_{e}\;}{q_{e}}\;=\frac{C_{e}}{q_{m}}+\;\frac{1}{K_{l}q_{m}} \end{array}$$

Where C_e_ indicates the concentration of metal ions in a solution (mg/L) at equilibrium, q_e_ is amount of metal ions adsorbed at equilibrium time (mg/g), q_m_ represent the maximum adsorption capacity in mg/g and K_L_ is Langmuir adsorption constant (Yagub et al., 2014). The Langmuir adsorption isotherm can be plotted between C_e_/q_e_ vs. C_e_, the straight line obtained from the data represent the fitness of the model and 1/q_m_ is the slope and 1/K_L_q_m_ is the intercept.

### Freundlich Adsorption Isotherm

To describe adsorption equilibrium, Freundlich equation is mostly used. This isotherm is used to describe adsorption of a large number of molecules on different adsorbents. When multilayer formation takes place, this isotherm is applicable on adsorption on heterogeneous surfaces (Ullah et al., 2017). This model is represented as follows:

$$\begin{array}{l} \ln\;q_{e}\;=\ln\;K_{f}+\;\frac{1}{n_{f}}\ln\;C_{e} \end{array}$$

Where q_e_ indicates the amount of metal ions that adsorbed at equilibrium, K_f_ denotes Freundlich constant and 1/n_f_ represents the heterogeneity factor. A favorable adsorption mainly hasa Freundlich constant n between 1 and 10. A strong interaction occurs between heavy metals and adsorbent when value of n is large. When 1/n is equal to 1 then it indicates linear adsorptions.

Both the models were compared in Table 3 which indicates the values of different parameters; R_L_, q_m_, n and Langmuir and Freundlich constants and their regression coefficient (*R*^2^). The value of n is >1 which indicated the favorable adsorption of metal ions on SF/bentonite adsorbent. The comparison of regression coeffient values showed that the Langmuir equation better explain the adsorption of heavy metals on SF/bentonite composite. These results also showed the homogeneity of adsorption surface of the SF/bentonite composite for adsorption of Cd(ll), Pb(ll), Hg(ll), and Cr(VI).

**Table 3:** The Langmuir and Freundlich adsorption isotherm parameters.

**Adsorbent**	**Metal ions**	**Calc. q_**m**_(mg/g)**	**Exp. q_**m**_(mg/g)**	**Langmuir K_**L**_(L/mg)**	***R*^**2**^**	**R_**L**_**	**n**	**Freundlich K_**f**_ (mg/g)**	***R*^**2**^**
SF/bentonite	Cd(II)	12.23	11.35	1.32	0.995	0.02	2.7	5.87	0.946
SF/bentonite	Pb(II)	11.8	11.1	1.44	0.988	0.022	3.36	5.92	0.990
SF/bentonite	Hg(II)	11.23	10.5	1.41	0.989	0.02	4.4	6.41	0.954
SF/bentonite	Cr(VI)	10.36	10.2	2.01	0.985	0.016	3.9	5.81	0.972

Where R_L_ is the separation factor and is calculated by using formula:

$$\begin{array}{l} R_{L}=\frac{1}{1+\;K_{L}C_{0}} \end{array}$$

The value of R_L_ shows the favorability of adsorption (Lin and Juang, 2002). In this equation C_o_ indicates the higher value of initial concentration of the solute. If the value of R_L_> 1 then isotherm is unfavorable, R_L_ = 1 indicates isotherm to be linear and R_L_ <1 indicates the favorability of isotherm. In this study, the values of R_L_ ranges between 0 and 1 that shows the favorability of the Langmuir adsorption process. For Cd(ll), Pb(ll), Hg(ll), Cr(VI) values of R_L_ were determined the higher values obtained at lower concentration confirmed that adsorption was favorable at lower concentrations.

### Kinetic Studies

Adsorption kinetics are used to explain the adsorption mechanism and characteristics of adsorption. Mostly the mechanism and rate of adsorption were studied by using pseudo first-order and pseudo second-order kinetic models.

#### Pseudo First-Order Reaction Kinetics

This model was proposed by Lagergren and Ho (Tomul and Basoglu, 2010). This was used for adsorption of heavy metals from aqueous solution. To calculate the rate constants for cadmium, lead, and mercury first order kinetics were used. These constants are calculated by using this equation:

$$\begin{array}{l} \text{log}\left(q_{e}-\;q_{t\;}\right)=\text{log }q_{e}-\frac{k_{1}}{2.303}\;t \end{array}$$

Where q_t_ (mg/g) is the amount of adsorption at time t and q_e_ (mg/g) represents the adsorption at equilibrium. And k_1_ the first order rate constant at equilibrium. The plot between ln (q_e_-q_t_) and time gives a straight line that indicates the first order kinetics is favorable. The value of adsorption rate constant k_1_ and values of q_e_ can be calculated from slope and intercept by plotting log (q_e_-q_t_). The process of adsorption is fast at initial stages and within 60 min it gained the equilibrium. The values of k_1_ calculated for Cd, Pb, Hg and Cr are 0.033, 0.028, 0.042, and 0.023 min^−1^, respectively. The suitably large capability and rapid kinetics for adsorption of these heavy metals on silk/clay composites indicated that it retains capacity to eradicate heavy metals from industrial wastewater as well.

#### Pseudo Second-Order Reaction Kinetics

To evaluate adsorption data pseudo second-order kinetics is also used. This reaction kinetics was proposed by Ho and McKay. This equation is represented as:

$$\begin{array}{l} \frac{dq_{t}}{dt}=\;k_{2}\;{\left(q_{e}-\;q_{t}\right)}^{2} \end{array}$$

Where k_2_ represents second order rate constant. When the above equation is integrated following expression is attained:

$$\begin{array}{l} \frac{t}{q_{t}}=\frac{1}{\left(k_{2}q_{e^{2}}\right)}+\;\frac{t}{q_{e}} \end{array}$$

By plotting t/qt vs. t, a straight line is obtained whose intercept is 1/k_2_${\text{q}}_{\text{e}}^{2}$ and slop is 1/q_e_. From intercept value of k_2_ is calculated and from slop value of q_e_ is calculated. Kinetic parameters of pseudo first order and pseudo second order are mentioned in Table 4. The regression coefficient (*R*^2^) values of pseudo-second order kinetic model are better than pseudo first order. In pseudo second order the value of regression coefficient was found good for all the metal ions and the calculated values of maximum adsorption capacity are closer to the experimental values. Hence, pseudo second order model fits very well with kinetic data as compare to pseudo first-order.

**Table 4:** The Pseudo-first and Pseudo-second order kinetic parameters for adsorption of Cd (ll), Pb (ll), Hg (ll), and Cr (VI) by SF/clay composite.

**Adsorbent**	**Metal ions**	**qe_**(exp)**_ (mg/g)**	**q_**e(cal.)**_ (mg/g)**	**Pseudo-first order K_**1**_(min^**−1**^)**	***R*^**2**^**	**q_**e(calc.)**_ (mg/g)**	**Pseudo second order K_**2**_ (mg/g)**	***R*^**2**^**
SF/bentonite	Cd(II)	4.4	2.45	0.033	0.945	4.44	0.02	0.977
SF/bentonite	Pb(II)	4.4	2.23	0.028	0.980	4.25	0.036	0.994
SF/bentonite	Hg(II)	4.35	2.6	0.042	0.967	4.64	0.026	0.985
SF/bentonite	Cr(VI)	4.45	2.21	0.023	0.980	4.07	0.04	0.996

## Conclusions

In this study, bentonite was selected as a low-cost adsorbent to make composites with silk fibroin to enhance its sorption efficiency for heavy metal ions. Formation of composite was confirmed by using FTIR and XRD analysis. Surface area and morphology of SF/clay composite was determined by BET and SEM analysis. The formation of SF/Clay composite possibly yielded mixture of random coils and β-sheets staked randomly along with the clay sheets. After modification and dispersion with clay, an increase in β-sheets content is expected which provide more room to chelate with heavy metals. Adsorption of heavy metal ions Cd(II), Pb(II), Hg(II), and Cr(VI) was studied by using batch experimental technique and it was observed that >90 percent of heavy metal ions removal was achieved by using 0.05 gram of SF/clay composite for lower initial metal ion concentration in a solutions. The adsorption of heavy metal ions was changed by changing pH, temperature and concentration. As the kinetic study was concerned, adsorption data fitted best to a pseudo second order kinetic model. The R_L_ values indicated that SF/clay composite was favorable for adsorption of heavy metal ions. In order to study the reaction equilibrium, Langmuir adsorption isotherm and Freundlich adsorption isotherms were studied. But the equilibrium data fitted best into Langmuir adsorption isotherm that shows the homogeneity of adsorbent sites. SF and clay both are natural occurring green, renewable and environmentally friendly resources and could be alternative to synthetic non-degradable materials using expensive technologies to build use and dispose them off.

## Data Availability Statement

The datasets generated for this study are available on request to the corresponding author.

## Author Contributions

AW and MSal developed the concept and designed the experiment. NW conducted the experiments. NW and MI wrote the first draft of the manuscript, MZ, MSae, and AM helped in conducting experiments. All authors listed have made a substantial, crucial and direct contribution to the work, and approved it for publication.

### Conflict of Interest

The authors declare that the research was conducted in the absence of any commercial or financial relationships that could be construed as a potential conflict of interest.
